# Nurses valued domains of living: Exploring gender differences

**DOI:** 10.1371/journal.pone.0307070

**Published:** 2024-08-13

**Authors:** Mohammed Munther AL-Hammouri, Jehad A. Rababah, Jorn Dormans

**Affiliations:** 1 Faculty of Nursing, Jordan University of Science and Technology, Irbid, Jordan; 2 Center for International Cooperation, Vrije Universiteit Amsterdam, Amsterdam, Netherlands; Alexandria University Faculty of Nursing, EGYPT

## Abstract

This study explores gender-specific aspects of nurses’ valued domains of living, examining differences in importance and consistency between male and female nurses in Jordanian hospitals. A cross-sectional study recruited 206 nurses (103 females, 103 males) from two major hospitals in Jordan. Data were collected using a demographics questionnaire and the Valued Living Questionnaire (VLQ), assessing 10 domains of living. The mean age of the sample was 35.4, ranging between 27 and 59 years old. The highest rank order valued domain of living for females was family, with 88.3% of females considering it a very important living domain. Parenting was the highest-ranked domain of living for males, with 76.7% reporting it as a very important domain of living. Significant gender differences were found in valued domains’ rank order, importance, and consistency. Female nurses prioritized work, education/training, spirituality, and citizenship/community more than males, while males prioritized marriage/couples/intimate relations. Females consistently aligned actions with values related to work, education/training, spirituality, and citizenship/community. Parenting emerged as a problematic valued domain, particularly for females. Understanding gender differences in nurses’ valued domains is essential for creating equitable and supportive work environments. Tailored interventions should address gender-specific needs and challenges, promoting workplace satisfaction and improving patient outcomes. Future research should explore factors contributing to gender differences and evaluate interventions promoting gender equity and diversity in nursing.

## Introduction

Nursing goes beyond just hospitals and clinics. It involves a complex mix of personal and professional values, beliefs, and areas of expertise that influence nurses’ lives [1]. These valued domains of living reflect how nurses’ personal experiences, priorities, and difficulties affect both their own well-being and the quality of care they give to patients, influencing how nurses deliver patient-centered care [2, 3]. Understanding these valued domains is important areas is vital in promoting a comprehensive approach to nursing, acknowledging that nurses’ personal experiences play a significant role in shaping healthcare settings [4].

Examining gender role within the context of nurses’ valued domains of living is crucial [5]. Studies have demonstrated that gender, as a social construct, impacts different aspects of human beings’ lives, job choices, family roles, and social roles [6, 7]. However, there is a significant gap in our knowledge within the nursing field regarding how gender relate to the valued domains of living [5]. Exploring gender differences helps us understand what influences their lives in the field, directing researcher, managers, and staff nurses where support, encouragement, and opportunities for growth can be customized according to gender [7, 8].

The importance of investigating gender differences in nurses’ valued domains is evidenced by the growing literature suggesting that understanding these differences can promote job satisfaction, decrease burnout, and lead to better quality of care and patients’ outcomes [9–11]. As nursing progresses, it’s important to acknowledge and tackle the distinct challenges that different genders encounter, fostering inclusive and supportive workplaces within the field [12]. Thus, this manuscript aims to explore and analyze the gender-specific aspects of nurses’ valued domains of living, providing a comprehensive perspective that contributes to the body of knowledge on gender differences within the nursing profession. Through this exploration, we aim to inform policy, practice, and education, fostering a more equitable and resilient nursing workforce.

The Acceptance and Commitment Therapy model (ACT) guided the current study. In Acceptance and Commitment Therapy (ACT), personal values are the cornerstone of the therapeutic approach, essential for guiding specific behavioral changes and fostering a healthy and meaningful life [13]. Given that values are proposed as a central mechanism of change, it is crucial to deeply understand the therapeutic valuing process that drives these targeted changes [13]. Additionally, considering the importance of gender in determining and understanding these values can further enhance the promotion of healthy and adaptive behavior. Although valued domains of living were rarely explored among nurses, previous studies showed significant gender differences in valued professional domains [5]. Thus, understanding gender differences is very crucial in planning and adapting to the work environment. The current research can improve patient care by fostering environments that support nurses’ well-being and professional growth. These insights can inform clinical decision-making by highlighting the need for personalized approaches in nurse management. Additionally, the findings can advance nursing education and policy by advocating for gender-sensitive training programs and workplace policies, ultimately leading to a more inclusive and effective nursing practice.

## Methods

### Design and setting

We used a cross-sectional design to achieve the current study goal. The data were collected from two major hospitals in Jordan’s central and northern regions between September 2022 and January 2023. The hospitals’ capacities range between 400 and 600 beds, serving most medical and surgical cases. Thus, they were considered high-end health services, meaning they provided most of the services unavailable in the peripheral hospitals and healthcare centers.

### Sample

We used a stratified random sample to recruit participants in the current study. Stratified random sampling is a powerful method to ensure adequate representation of different subgroups within the population. By stratifying the sample based on gender, we effectively accounted for potential differences between male and female nurses, leading to more accurate and generalizable findings. G*Power software was utilized to establish the minimum required sample size for a two-group t-test, considering parameters of a small to medium effect size (0.35), a significance level of 0.05, and a power of 0.8. The required sample size was 204 based on these parameters. The inclusion criteria were at least six months of experience in the current nursing position, being married, and having at least one kid. The reason for the latter criterion is that some values were asking about parenting, and we wanted to make sure that all values applied to our participants. The sample was recruited in two phases: phase to collect the data from a minimum of 102 female nurses, and phase 2 to collect the data from male nurses matching the number of female nurses.

A research assistant collected the data in the current study. He met with potential participants and invited them to participate. Those who agreed received a detailed explanation of the study and were handed the study questionnaires to complete and return. The participants completed the paper-based questionnaires after signing the informed consent.

### Measures

A demographics questionnaire was developed for the current study to collect demographic characteristics of our sample, such as gender, age, and highest academic degree. The valued living questionnaire was used to gather data about nurses’ valued domains of living importance and consistency of actions to these valued domains of living.

### Valued domains of living

Valued domains of living were assessed using the Valued Living Questionnaire (VLQ). The VLQ consists of 10 living domains: Family, marriage/couples/intimate relations, parenting, friendship, work, education, recreation, spirituality, citizenship, and physical self-care [14]. The VLQ consists of two parts. The first part asks the participants to rate each of the aforementioned valued domains of living on a rating scale from 1 to 10, with 1 indicating absence of importance and 10 indicating extreme importance. The second section, similarly, asks about the same domains of valued living, except it asks about how the participants think their actions during the last were consistent in correspondence with each value [14], with 1 indicating complete inconsistency of the actions with the corresponding valued domain of living, and 10 indicating complete consistency. The VLQ showed good test-retest reliability [14]. Values that are scored as 9 or 10 are considered highly important. The developer suggests that valued domains of living scored high in importance (scored as 9 or 10) combined with a score of 6 or below on the consistency scale are problematic and require special attention [14]. The reason is that these valued domains of living are seen as extremely important by the participants. Yet, the daily actions are inconsistent with them, which may lead to serious issues that will be discussed in more detail in the discussion section. The VLQ is reported as a valid and reliable measure of the valued domain of living [14].

### Data analysis

In the current study, we used SPSS version 26 to analyze the data to achieve the study goal. Initially, we examined the sample basic demographic characteristics of the study sample. We categorized the importance scale scores into two categories to compare the rank order of the valued domain of living according to the measure developer. The values scored as 9 and 10 are considered high priority (high importance according to the measure developer), while all other scores are categorized as lower priority. Then, the frequencies of participants scoring the value as a high priority for each gender were calculated (see results section). Then, for each gender, the values were ranked based on the number of participants who scored them as a high priority. Then, the mean scores for each value were used to compare males and females regarding the importance of these valued domains of living and the consistency of actions with the valued domains of living.

According to the measure developer, the most concerning values (problematic valued domains of living) are those with high priority (scores 9 and 10 on the importance part) and low consistency scores (scores of 6 and below on the consistency scale). Thus, we similarly dichotomized the valued domains of living accordingly to high (score 7 and above) and low consistency (score 6 or below) domains of living. Then, we categorized the data for each gender to explore the number of participants who meet these two criteria for both genders under each value. We used chi-square to examine if there was a significant difference in the frequencies of participants with high importance and low consistency between males and females under each valued living domain.

### Ethical considerations

The Institutional Review Board’s approval was obtained before data collection from Jordan University of Science and Technology. An experienced research assistant collected the data. The eligible participants were approached and invited to the study. Those who agreed to participate signed a written informed consent after the study was explained to them before completing the questionnaires. The participants were informed that they could withdraw from the study without consequences. The data collected from the participants did not have any identifiable data. The participants were given the primary investigator’s contact in case they had any questions.

## Results

### Sample characteristics

We recruited a sample of 206 nurses (103 females and 103 males). The average age for our sample was 35.4 (SD = 7.2). The majority have a bachelor’s degree and are married (Table 1)

**Table 1:** Participants’ characteristics (N = 206).

Variable	n	~Percentage
**Gender**		
Females	103	50
Males	103	50
**Highest Educational Level: **		
Bachelor	181	87.9
Master’s	25	12.1
**Marital Status**		
Married	201	97.6
Separated	5	2.4
	**M (SD)**	**Range**
**Age (years) **	35.4 (7.2)	27–59

### Value rank order

As explained in the data analysis, the frequencies of participants’ scored values as a high priority were used to rank order valued domains of living comparing males and females (Table 2). The results showed some fundamental differences across the rank order of the values. For example, the rank order of the work as a value was three rank orders higher for females than males. This means that work tends to be more important to females than males (Table 2). On the other hand, marriage was four rank orders higher for males than females, meaning that males tend to see marriage/couples/intimate relations as more important than females.

**Table 2:** Values rank order based on the frequency of participants scored the values as high priority (scored 9 or 10 on the value importance).

Rank	Females		Males	
	Value(s)	Frequency of participants	Value(s)	Frequency of participants
1	• Family (other than marriage or parenting)	91	• Parenting	79
2	• Parenting	85	• Family (other than marriage or parenting)	75
3	• Spirituality	84	• Spirituality	74
4	• Physical self-care (diet, exercise, sleep)	72	• Marriage/couples/intimate relations	71
5	• Education/training	70	• Recreation/fun	61
	• Work			
6	• Education/training	70	• Physical self-care (diet, exercise, sleep)	56
	• Work			
7	• Recreation/fun	68	• Citizenship/Community Life	54
8	• Friends/social life	62	• Education/training	53
	• Marriage/couples/intimate relations			
9	• Friends/social life	62	• Friends/social life	48
	• Marriage/couples/intimate relations		• Work	
10	• Citizenship/Community Life	59	• Friends/social life	48
			• Work	

### Independent sample t-test

An Independent sample t-test was used to examine the difference in mean scores of importance and consistency of the valued domains of living (Tables 3 and 4). For values importance scores, the independent sample t-test showed significant differences between female and male nurses in Education/Training, Spirituality, Citizenship/Community, and Family valued domains of living. The examination of the mean scores for females and males showed that females tended to score higher in importance of the values of Family, Education/Training, Spirituality, and Citizenship/Community. This means that females see these valued domains of living as more important than males.

**Table 3:** Independent samples t-test-value importance (N = 206).

Predictor	*t*	*p*	Mean Difference (F—M)	LLCI	ULCI
Family (other than marriage or parenting)	2.46	.015	0.73	0.14	1.31
Marriage/couples/intimate relations	-1.40	.16	-0.47	-1.14	0.19
Parenting	0.90	0.37	0.27	-0.32	0.86
Friends/social life	1.13	0.26	0.33	-0.24	0.90
Work	0.26	0.79	0.25	-1.65	2.16
Education/training	2.50	0.01	0.72	0.15	1.28
Recreation/fun	1.89	0.06	0.56	-0.02	1.15
Spirituality	2.26	0.02	0.67	0.09	1.25
Citizenship/Community Life	2.28	0.02	0.79	0.11	1.48
Physical self-care (diet, exercise, sleep)	1.88	0.06	0.54	-0.03	1.11
**Variable(s)**	**Gender**	**Mean**		**SD**	
Family (other than marriage or parenting)					
	Females	8.98		1.69	
	Males	8.25		2.48	
Marriage/couples/intimate relations					
	Females	7.65		2.53	
	Males	8.13		2.27	
Parenting					
	Females	8.89		2.19	
	Males	8.62		2.11	
Friends/social life					
	Females	7.68		2.08	
	Males	7.35		2.10	
Work					
	Females	8.06		1.69	
	Males	7.80		1.68	
Education/training					
	Females	8.13		1.77	
	Males	7.41		2.31	
Recreation/fun					
	Females	8.09		1.88	
	Males	7.52		2.36	
Spirituality					
	Females	8.76		1.89	
	Males	8.76		2.30	
Citizenship/Community Life					
	Females	7.73		2.19	
	Males	6.93		2.75	
Physical self-care (diet, exercise, sleep)					
	Females	8.19		1.92	
	Males	7.65		2.22	

LLCI: Lower Limit Confidence Interval; ULCI: Upper Limit Confidence Interval

**Table 4:** Independent samples t-test-value consistency (N = 206).

Predictor	*t*	*p*	Mean Difference (F—M)	LLCI	ULCI
Family (other than marriage or parenting)	1.30	0.19	0.41	-0.21	1.02
Marriage/couples/intimate relations	-1.89	0.06	-0.68	-1.39	0.03
Parenting	-0.95	0.34	-0.34	-1.04	0.36
Friends/social life	1.63	0.11	0.55	-0.12	1.21
Work	3.36	< 0.001	1.16	0.48	1.84
Education/training	2.12	0.03	0.75	0.05	1.45
Recreation/fun	0.63	0.53	0.23	-0.48	0.94
Spirituality	2.31	0.02	0.76	0.11	1.39
Citizenship/Community Life	2.15	0.03	0.81	0.07	1.56
Physical self-care (diet, exercise, sleep)	1.54	0.12	0.52	-0.15	1.21
**Variable(s)**	**Gender**	**Mean**		**SD**	
Family (other than marriage or parenting)					
	Females	8.01		2.02	
	Males	7.60		2.45	
Marriage/couples/intimate relations					
	Females	6.62		2.56	
	Males	7.30		2.58	
Parenting					
	Females	7.35		2.65	
	Males	7.69		2.38	
Friends/social life					
	Females	6.82		2.38	
	Males	6.27		2.44	
Work					
	Females	7.63		1.95	
	Males	6.47		2.88	
Education/training					
	Females	6.95		2.32	
	Males	6.19		2.71	
Recreation/fun					
	Females	6.47		2.54	
	Males	6.24		2.64	
Spirituality					
	Females	7.82		1.98	
	Males	7.07		2.64	
Citizenship/Community Life					
	Females	6.72		2.47	
	Males	5.91		2.91	
Physical self-care (diet, exercise, sleep)					
	Females	7.04		2.32	
	Males	6.51		2.59	

LLCI: Lower Limit Confidence Interval; ULCI: Upper Limit Confidence Interval

Concerning consistency between valued domains of living and actions over the last week, the results showed significant differences between females and males in terms of consistency between last week’s action and valued domain of living in terms of Work, Education/Training, Spirituality, and Citizenship/Community. Similarly, the examination of the mean scores showed that females tended to score significantly higher on consistency in Work, Education/Training, Spirituality, and Citizenship/Community. This means females’ actions tended to be more consistent with their valued domains of living in these domains than males.

### Problematic valued domains of living

The developer of the VLQ stressed that the most worrying (problematic) valued domains of living are those the participants score as highly important (scored as 9 or 10) and, simultaneously, scored low in consistency (scores of 6 or below) on the consistency part. To explore the presence of such a combination, the frequencies of this combination were examined for the total sample, female and male participants (Table 5). The Chi-square analysis was used to examine if the frequencies of participants who showed problematic versus nonproblematic valued domains of living differed based on gender. The chi-square results showed that there was a significant relationship only between gender and parenting (Chi-square (1, 206) = 4.72, p = .03). Looking at the frequencies (Table 5), this result indicates that more females tended to give the Family valued domain of living high importance. At the same time, their actions were significantly inconsistent compared to males.

**Table 5:** Problematic values: Scored high on importance (9 or 10) and low on consistency (below 6).

Value	Frequencies		
	Total	Female(s)	Male(s)
• Family (other than marriage or parenting)	19	9	10
• Marriage/couples/intimate relations	19	12	7
• Parenting	24	17	7
• Friends/social life	16	10	6
• Work	8	5	3
• Education/training	19	9	10
• Recreation/fun	26	11	15
• Spirituality	17	7	10
• Citizenship/Community Life	13	6	7
• Physical self-care (diet, exercise, sleep)	19	7	12

## Discussion

The present study offers valuable insights into the gender-specific aspects of nurses’ valued domains of living, highlighting variations in the importance and consistency of valued domains of living across genders. Our study reveals significant variations in how male and female nurses prioritize and align their own actions with various valued domains of living, signifying the need for tailored approaches to support and empower nurses based on their gender-specific valued domains of living. Although the area of gender differences concerning valued domains of living was not explored earlier among nurses, these results are consistent with similar studies that have found significant gender differences in terms of professional values [5].

One notable finding from our study is the distinct rank order of valued domains of living between male and female nurses. Females prioritize Work, Education/Training, Spirituality, And Citizenship/Community more than males, while males prioritize Marriage/Couples/Intimate Relations more than females. This suggests that gender plays a crucial role in shaping the perceived importance of different aspects of life among nurses. Understanding these differences can inform targeted interventions and support mechanisms to enhance workplace satisfaction and well-being among nurses. Valuable interventions, such as value-driven approaches, have been introduced in the literature and linked with promoting human beings’ well-being [15], and used to plan and develop tailored interventions according to prioritized valued domains of living. Different interventions, including those based on acceptance and commitment therapy, showed superior impact to other various interventions when it was values-based [16].

Consistency between actions and valued domains of living is another crucial aspect explored in our study. Females tend to exhibit higher consistency than males in aligning their actions with values related to Work, Education/Training, Spirituality, And Citizenship/Community. This finding underscores the importance of promoting congruence between personal values and professional behaviors, particularly among female nurses who may face unique societal and organizational pressures. This notion is essential to consider since the inconsistency between actions and behaviors and valued domains of living can lead to cognitive dissonance, leading to serious psychological consequences [17, 18]. Such a phenomenon in motivational interviewing is referred to as developing discrepancy [19–21]. This process involves increasing individuals’ awareness of the conflict between their behaviors and their values. Highlighting this inconsistency is a critical step in guiding individuals toward a state where their behaviors are more aligned with their values, thereby reducing the psychological impact of the discrepancy [17, 18].

Furthermore, our analysis identified problematic valued domains of living characterized by high importance but low consistency with personal actions, particularly the Parenting domain of living. This discrepancy is more pronounced among female nurses, indicating potential challenges in balancing family responsibilities with their professional duties. Addressing these challenges requires specialized interventions to support nurses, such as flexible work conditions, childcare assistance, and access to resources to promote work-life balance. Such a problematic valued domain of living can be simply a product of a conflict between nurses’ valued domain of living and work-related values and priorities [22].

The implications of our findings extend beyond individual nurses to encompass broader organizational and policy considerations within the nursing field. Creating inclusive, supporting, and empowering work environments requires an understanding of gender-related differences in valued domains of living and a commitment to addressing the unique needs of different genders. Strategies to promote gender equity and diversity in nursing should focus on actions that build an environment where all nurses feel respected, valued, and supported, no matter their gender. To effectively address diversity and gender differences among individuals, it’s important to consider their personal values, ensuring that interventions are planned in a way that respects individual differences and is tailored to meet their needs [15].

### Relevance to the clinical practice

Overall, recognizing and addressing gender differences in nurses’ valued domains of living is essential for creating inclusive, equitable, empowering, and supportive work environments. By implementing targeted interventions at the organizational, policy, educational, and advocacy levels, stakeholders can work together to address gender differences to ultimately improve the quality of care provided.

From the practical point of view, a logical step for the current study findings is to examine the role of the profession, organization, education, and other work or professional factors that contribute to such gender differences or problematic domains of living. This will open the door for future recommendations to address any associated issues using value-based interventions [15].

### Limitations

While our study provides valuable insights into gender differences in nurses’ valued domains of living, certain limitations should be acknowledged. The cross-sectional design limits our ability to infer causality or temporal relationships between variables. Additionally, the study sample was drawn from specific hospitals in Jordan, which may limit the generalizability of our findings to other contexts or populations. Another challenge is the absence of similar studies that examined this area in nurses, limiting our ability to make more in-depth analyses and syntheses based on previous research.

Future research could employ a longitudinal design to capture temporal changes and causal relationships in nurses’ valued domains of living. Additionally, expanding the sample to include nurses from diverse geographic regions and various types of healthcare settings would enhance the generalizability of our findings. These modifications would provide a deeper understanding of gender differences in nurses’ valued domains and strengthen the validity of our conclusions.

## Conclusion

In conclusion, our study underscores the importance of recognizing and addressing gender differences in nurses’ valued domains of living to promote a more equitable and supportive nursing workforce. By understanding the unique needs and challenges different genders face, healthcare organizations and policymakers can implement targeted interventions to promote workplace satisfaction and retention rates and ultimately improve quality of care. Future research should continue to explore gender dynamics in nursing and evaluate the effectiveness of interventions promoting gender equity and diversity in the profession.
